# Switching to bedaquiline for treatment of rifampicin-resistant tuberculosis in South Africa: A retrospective cohort analysis

**DOI:** 10.1371/journal.pone.0223308

**Published:** 2019-10-17

**Authors:** Tara C. Bouton, Margaretha de Vos, Elizabeth J. Ragan, Laura F. White, Leonie Van Zyl, Danie Theron, C. Robert Horsburgh, Robin M. Warren, Karen R. Jacobson

**Affiliations:** 1 Division of Infectious Diseases, Brown University Alpert School of Medicine, Providence, RI, United States of America; 2 Department of Science and Technology, National Research Foundation Centre of Excellence in Biomedical Tuberculosis Research, South Africa Medical Research Council for Tuberculosis Research, Cape Town, South Africa; 3 Division of Molecular Biology and Human Genetics, Faculty of Medicine and Health Sciences, Stellenbosch University, Cape Town, South Africa; 4 Section of Infectious Diseases, Boston University School of Medicine, Boston, MA, United States of America; 5 Department of Biostatistics Boston University School of Public Health, Boston, MA, United States of America; 6 Brewelskloof Hospital, Worcester, South Africa; 7 Department of Medicine, Boston University School of Medicine, Boston, MA, United States of America; 8 Departments of Epidemiology, Biostatistics and Global Health, Boston University School of Public Health, Boston, MA, United States of America; Jamia Hamdard, INDIA

## Abstract

South Africa led the world with guidelines on bedaquiline (BDQ) use as a single drug substitution to manage rifampin resistant tuberculosis regimen toxicity. We examined reasons for giving BDQ in a retrospective cohort: >75% of patients were switched to BDQ for toxicity (ototoxicity or renal dysfunction) rather than drug resistance.

## Background

Patients with rifampin-resistant tuberculosis (RR-TB) often have baseline resistance to additional drugs in the second-line TB regimens [1] and as many as 80% develop side effects [2] (such as hearing loss from injectables (SLI)), leading to global demand for new agents. In June 2015, South Africa became one of the first countries to use newly-available bedaquiline (BDQ) for single drug substitution to manage second-line drug toxicity [3], as long as the patient was not failing current therapy. Single drug substitution is only reasonable if a patient has a strong regimen background and the new drug is being introduced for toxicity reasons. If a patient is failing therapy then at least two new effective drugs should be introduced to avoid resistance acquisition. Whether South Africa’s BDQ implementation strategy would balance the need for a less toxic drug to prevent further patient disability with international calls for strict TB drug stewardship to mitigate risk of BDQ introduction into weak regimens is not known [4].

BDQ’s long half-life (6 months) may make it particularly vulnerable to resistance acquisition [5], especially in settings with high treatment loss to follow-up. Emergence of BDQ resistance is well documented [4]. In one study, BDQ resistance-associated variants were found in 6.3% of BDQ-naïve multidrug-resistant (MDR) TB isolates, suggesting a role of prior TB drug exposure [6] and possibly transmission of drug-resistant strains. Nonetheless, in August 2018, supported by a meta-analysis [7] and observational studies [8], the WHO recommended including BDQ as a group A drug in long course MDR-TB regimens [9]. With this change, evaluation of programmatic use of BDQ is urgently needed.

We retrospectively examined the indications for BDQ introduction to patients’ regimens and available resistance test results at the time of BDQ initiation among a cohort of South African patients being treated for RR-TB.

## Methods

We conducted a retrospective cohort study of all RR-TB patients at Brewelskloof Hospital, Worcester, South Africa who initiated treatment from December 2015 through June 2017. Eligible patients had evidence of RR-TB by genotypic or phenotypic drug susceptibility testing (DST) and were ≥18 years old. Phenotypic DST was performed until December 2016 and genotypic DST was used from January 2017 onwards. Brewelskloof Hospital is the regional referral inpatient TB facility and RR-TB care is practiced according to South African National Treatment Program tuberculosis guidelines [10]

During the study period, an 18–24 month RR-TB regimen was administered with SLI for the initial 6–8 months. Additional drugs included in the regimen were high dose isoniazid, pyrazinamide, ethambutol, ethionamide, terizidone and moxifloxacin (levofloxacin was substituted where BDQ was added). South African national guideline-approved indications for the switch to BDQ included: additional drug resistance (fluoroquinolone (FQN), SLI, or both *inhA* promoter and *katG* gene mutations), moderate to severe toxicity due to second line agents (e.g., hearing loss or renal dysfunction), or history/candidate for pneumonectomy/lobectomy [10]. In September 2015, the Western Cape Department of Health (DOH) expanded drug access and BDQ applications were reviewed by the provincial or national clinical advisory committee prior to medication provision. If FQN/SLI resistance was detected, patients received an individualized regimen based on DST testing potentially containing the additional drugs, PAS, linezolid or clofazimine. BDQ was dosed according to the South African national guideline: 400mg daily for 2 weeks, then 200mg daily on Monday, Wednesday and Friday for 22 weeks. Continuation of BDQ beyond 6 months required clinical advisory committee approval.

Data were abstracted from TB registers, laboratory registers, and EDRweb.net, along with detailed review of patient hospital records, including: medication administration records, audiometry, provincial/national committee BDQ applications, and clinical notes. In addition to the guideline-approved indications for switch to BDQ described above [10], two additional committee approved indications were noted in chart review. In one case, the patient’s age was thought to put them at increased risk for SLI intolerance, and in two cases clinical RR-TB regimen failure (persistent culture positivity without additional drug resistance on DST) were approved as indications for switching to a BDQ-containing regimen (the regimens were further individualized in the clinical failure cases). Study data were collected and maintained in REDCap (https://projectredcap.org/).

We compared baseline characteristics between patients treated with and without BDQ using Pearson chi-squared, Fisher’s exact test, and Student t-tests where appropriate. Among those treated with BDQ, we then analyzed the indications for switching, available DST, and additional chemotherapeutic agents added at the time of BDQ initiation. Analysis was completed using SAS version 9.4.

Ethical approval for this study was obtained from Boston University Medical Campus Institutional Review Board (IRB), the Miriam Hospital IRB, Stellenbosch University Human Research Ethics Council, and the Western Cape DOH Ethics Board. The requirement for informed consent was waived for this study.

## Results

**Fig 1:**
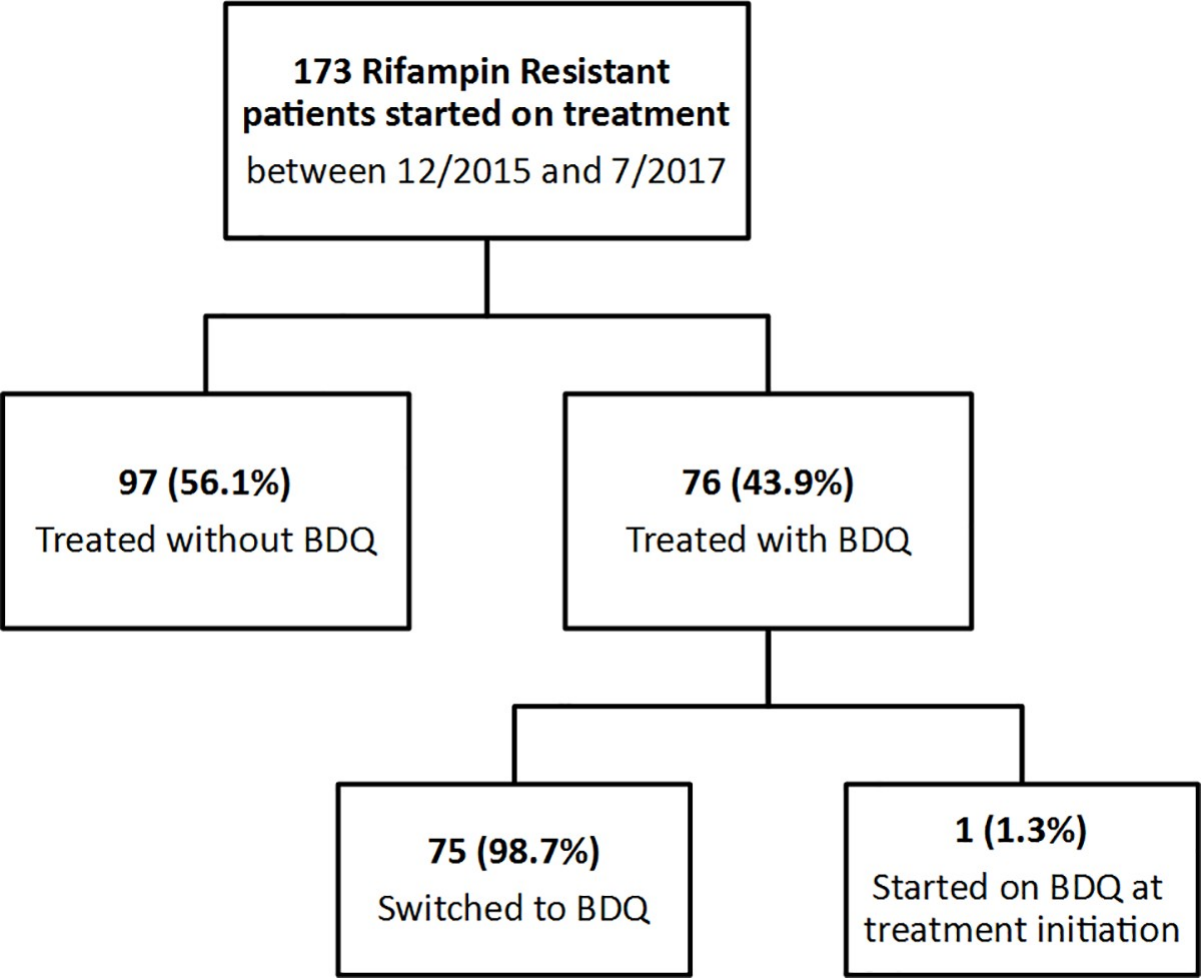
Flow diagram of rifampin resistant tuberculosis patients at brewelskloof hospital.

Of 173 RR-TB patients who initiated treatment, one patient (0.6%) who had prior treatment failure was initiated on an individualized BDQ-containing regimen and 75 (43.4%) were switched to a regimen with BDQ (Fig 1). Of all included patients, 62.4% were HIV co-infected (Table 1). Use of BDQ increased steadily during the study period. In the first 7 months of this cohort, 18.7% (14/75) of RR-TB patients received BDQ, which increased to 50.9% (28/55) during the following 6 months and finally to 79.1% (34/43) in the final 6 months. All patients were treated with at least five drugs. Compared to those who did not receive BDQ, patients who received BDQ were older (median 41.6 years vs 37.3, p = 0.0145, Table 1). There were no other significant differences in baseline demographic characteristics between the two groups (Table 1).

**Table 1:** Drug-resistant tuberculosis cohort characteristics by whether or not bedaquiline was included in the regimen.

	Total	Treated without Bedaquiline	Treated with Bedaquiline	p
	173	97 (56.1)	76 (43.9)	
Male (%)	91/173 (52.6)	52 (53.6)	39 (51.3)	0.76
Age, years (mean, SD)	39.1 (11.4)	37.3 (10.2)	41.6 (12.5)	0.01
Extrapulmonary disease (%)	39/173 (22.5)	18 (18.6)	21 (27.6)	0.16
At least 1 Prior TB Episode (%)	130/172 (75.6)	75 (78.1)	55 (72.4)	0.38
BMI (mean, SD)	19.01 (4.5)	18.85 (3.9)	19.18 (5.1)	0.65
HIV positive (%):	108/173 (62.4)	58 (59.8)	50 (65.8)	0.42

Abbreviations: SD, standard deviation; TB, tuberculosis; BMI, body mass index; HIV, human immunodeficiency virus.

The reason for BDQ introduction was additional drug resistance in 21.1% (16/76) of cases and intolerance of second line therapy in 76.3% of cases (58/76, Table 2), of which >70% was attributed to hearing loss. DST to SLIs and FQNs was completed at the time of switch to BDQ in 79.0% of patients (60/76, Table 2). Among those with DST available, 21.7% had SLI/FQN resistance detected (13/60, Table 2); additional drugs were added along with BDQ in all cases (Table 2). However, among the 16 patients for whom SLIs and FQN testing was unknown, the switch to BDQ was approved as a single drug substitution in 15/16 (93.8%, Table 2).

**Table 2:** Indications for the use of bedaquiline, second line drug susceptibility test results when bedaquiline was started, and the resulting changes in regimen among patients who received bedaquiline (n = 76).

	Total (%)	N (%)
Additional Drug Resistance (%):	n = 16 (21.1)	
∙ Both InhA and KatG		3/76 (3.9)
∙ Pre-XDR		11/76 (14.5)
∙ XDR		2/76 (2.6)
Intolerance of SL drugs (%):	n = 58 (76.3)	
∙ Hearing loss		41/76 (53.9)
∙ Renal Dysfunction		10/76 (13.2)
∙ Both renal and hearing		5/76 (6.6)
∙ Neuropathy		1/76 (1.3)
∙ Age		1/76 (1.3)
History/candidate for pneumonectomy/lobectomy (%):	n = 0 (0.0)	
Clinical Treatment Failure (%):	n = 2 (2.6)	
FQN/SLI testing:		
∙Available		60/76 (79.0)
∙ Not Available		16/76 (21.0)
FQN/SLI resistance unknown:	16/76 (21.0)	
∙ Additional drugs added		1/16 (6.3)
FQN/SLI resistance detected:	13/60 (21.7)	
∙ Additional drugs added		13/13 (100.0)

Abbreviations: additional drug resistance, resistance to drugs beyond rifampin and isoniazid; pre XDR, pre extensively drug resistant (resistant to either a second line injectable or fluoroquinolone); XDR, extensively drug resistant (resistant to both second line injectables and fluoroquinolones; SL, second line; FQN, fluoroquinolone; SLI second line injectable

## Discussion

In our retrospective cohort of patients hospitalized with RR-TB, BDQ use increased steadily during the 19-month study period. FQN and SLI susceptibility results were available in >75% of individuals switched to BDQ. All patients found to have FQN or SLI resistance had additional drugs added to strengthen their regimen at the time of BDQ switch. However, a small group of patients with unknown FQN/SLI susceptibility felt to be responding to treatment also received committee-approval for single drug substitution. Those who received BDQ were switched overwhelmingly for hearing loss from SLIs rather than additional drug resistance. Our findings of high rates of hearing loss with SLI treatment are consistent with other studies [11] and further support removing SLIs from first line MDR-TB therapy.

In September 2015, the Western Cape Provincial DOH expanded BDQ access. Providers could gain approval via a standardized application to give BDQ to patients who had adverse reactions to the standard regimen. In our cohort, <20% of RR-TB patients initiated on treatment between December 2015 and June 2016 were switched to BDQ compared to almost 80% of patients a year later. Early during the roll out at Brewelskloof Hospital, there was an episode of severe QTc prolongation attributed to BDQ, which may have led providers to be more cautious in prescribing the newer drug and instead continuing with the prior practice of SLI dose adjustment for toxicity. Our study lacked qualitative insight into clinician prescribing practices, but the trend indicates that with experience and lack of additional negative events, providers did readily change to the new medication option within a year of its introduction.

The South African programmatic Clinical Advisory Review Committee process was created to ensure that all patients with known FQN/SLI resistance were switched to an optimized BDQ regimen that includes additional effective drugs. Our data show this method of stewardship to be relatively effective. Globally, optimal use of and access to companion medications have been universal obstacles as countries roll out BDQ [3]. In settings like South Africa where routine second line DST is limited to FQN, SLI, and ethionamide (via mutations in the *inhA* promoter region on line probe assay) and with more than half of global MDR-TB cases estimated to be resistant to pyrazinamide [12], the strength of the BDQ backbone regimen is difficult to guarantee. A significant limitation of our study was the inability to assess whether BDQ resistance emerged. Under the previous WHO MDR-TB regimen recommendations, South Africa opted to allow for BDQ use as a single substitution for adverse reactions if the patient did not appear to be failing their regimen. Patients with a single substitution within a strong backbone regimen should not be at increased risk of acquiring resistance, but future work should monitor these patients to ensure this assumption is correct. Our work highlights that despite a well-developed national health laboratory system, there remain patients for whom SLI/FQN susceptibility is unknown, in addition to the drugs (pyrazinamide, ethambutol, terizidone) that are not tested. While lack of second line DST may have been due to early culture conversion in those patients, suggesting good clinical response to therapy, ongoing surveillance is needed to confirm that switching to BDQ remains safe and effective without leading to additional emergence of resistance.
